# Supplementary material for: A Likelihood Approach for Real-Time Calibration of Stochastic Compartmental Epidemic Models
Source: PLoS Comput Biol. 2017 Jan 17;13(1):e1005257. doi: 10.1371/journal.pcbi.1005257 (PMC5240920; doi:10.1371/journal.pcbi.1005257)
Supplement: S1 File — (TAR.GZ) [file pcbi.1005257.s014.tar.gz › HSPH_Online-SI-Revision/Additions/MSS11/n100-extreme_noisy/n100-extreme_noisy_NEW_table0.pdf]

1	R0	{2.76052, 16.3913, 0.1}	{2.05153, 9.97436, 1.22}	{{1.88486, 8.39294, 1.}, {2.36011, 13.1698, 2.}}	{27.2825, 41.6907, 1120.}	inf	26 580.	15 200.2	{12 428., 20 868.1}	44.7107	0.270478	newly	10 934.9	9801.77	{9468., 10 405.}	10.3623	0.0477503	Reff	0.831027	0.839772	{0.807784, 0.847851}	1.7009	ar	92 090.	79 290.4	{75 826., 87 606.}	14.2968
2	R0	{2.18202, 15.1734, 3.}	{2.22867, 15.6411, 3.}	{{2.22867, 15.6411, 3.}, {2.22867, 15.6411, 3.}}	{2.13793, 3.08213, 0.}	inf	17 999.	18 701.3	{18 548.7, 18 856.9}	3.90215	0.0166166	newly	8027.	8014.62	{7883., 8122.}	0.881276	0.00383258	Reff	0.873157	0.859703	{0.85704, 0.862515}	1.5409	ar	84 264.	84 930.5	{84 668., 85 176.}	0.79099
3	R0	{2.57891, 12.5971, 1.}	{2.53075, 11.6597, 0.}	{{2.53075, 11.6597, 0.}, {2.53075, 11.6597, 0.}}	{1.86748, 7.44128, 100.}	inf	22 711.	21 631.8	{21 450.1, 21 814.1}	4.75173	0.0211515	newly	11 421.	11 663.2	{11 550., 11 796.}	2.14009	0.00918183	Reff	0.727149	0.705178	{0.701859, 0.70855}	3.02154	ar	90 302.	89 654.1	{89 479., 89 835.}	0.717481
4	R0	{2.5856, 9.28508, 0.1}	{2.14326, 6.48739, 0.}	{{2.14326, 6.48739, 0.}, {2.14326, 6.48739, 0.}}	{17.1078, 30.131, 100.}	inf	23 144.	16 406.9	{16 237.9, 16 582.9}	29.1096	0.149429	newly	15 345.	15 081.2	{14 908., 15 256.}	1.77445	0.00779106	Reff	0.760942	0.778888	{0.774216, 0.783105}	2.35837	ar	90 312.	83 145.4	{82 877., 83 427.}	7.93534
5	R0	{2.4406, 12.1341, 1.}	{2.53075, 11.6597, 0.}	{{2.53075, 11.6597, 0.}, {2.53075, 11.6597, 0.}}	{3.69373, 3.90952, 100.}	inf	22 895.	23 789.5	{23 595.7, 23 957.7}	3.90714	0.0166375	newly	12 782.	13 821.1	{13 655., 13 989.}	8.12901	0.0339246	Reff	0.972189	1.00098	{0.997037, 1.00479}	2.96119	ar	88 597.	89 659.	{89 449., 89 844.}	1.19867
6	R0	{2.72155, 15.4094, 2.}	{2.31216, 11.5471, 0.2}	{{2.15677, 10.1363, 0.}, {2.93371, 17.1903, 1.}}	{18.1607, 29.6876, 90.}	inf	26 147.	19 824.7	{17 405.9, 28 861.5}	28.3275	0.146773	newly	11 271.	11 318.2	{11 131., 11 482.}	1.00639	0.00435524	Reff	0.880857	0.870331	{0.853566, 0.877542}	1.19495	ar	91 682.	85 467.5	{83 220., 93 546.}	7.60004
7	R0	{2.63781, 14.3935, 0.1}	{2.67666, 15.8766, 0.}	{{2.46794, 14.1192, 0.}, {2.96797, 18.4042, 0.}}	{7.28873, 12.0189, 100.}	inf	24 939.	25 019.4	{22 117.7, 29 027.9}	11.0424	0.0473905	newly	11 237.	10 429.2	{10 277., 10 581.}	7.18884	0.0324256	Reff	0.790499	0.817084	{0.784101, 0.835836}	3.68654	ar	91 192.	91 005.1	{88 707., 93 883.}	2.22547
8	R0	{2.82391, 16.104, 1.}	{2.15677, 10.1363, 0.}	{{2.15677, 10.1363, 0.}, {2.15677, 10.1363, 0.}}	{23.6246, 37.0574, 100.}	inf	27 368.	17 603.1	{17 369.5, 17 802.9}	35.6801	0.191672	newly	11 207.	11 367.1	{11 198., 11 511.}	1.51486	0.00651405	Reff	0.861801	0.873759	{0.869968, 0.877608}	1.38754	ar	92 664.	83 474.8	{83 160., 83 737.}	9.9167
9	R0	{2.80645, 14.6457, 0.1}	{1.9505, 8.69558, 0.}	{{1.93419, 8.56054, 0.}, {1.93419, 8.56054, 0.}}	{30.4992, 40.8092, 100.}	inf	27 606.	14 348.8	{13 924.8, 14 268.3}	48.0228	0.286407	newly	12 694.	11 008.3	{10 815., 11 127.}	13.2793	0.0619436	Reff	0.94945	0.951583	{0.946184, 0.954077}	0.446157	ar	92 567.	78 089.3	{77 439., 78 186.}	15.6402
10	R0	{1.86213, 5.4271, 0.1}	{2.80569, 11.6897, 1.}	{{2.80569, 11.6897, 1.}, {2.80569, 11.6897, 1.}}	{50.6709, 115.394, 900.}	inf	10 896.	25 750.2	{25 560., 25 950.7}	136.327	0.373506	newly	11 765.	13 795.5	{13 656., 13 930.}	17.2585	0.0691317	Reff	0.763157	0.710073	{0.703897, 0.714742}	6.95583	ar	75 362.	92 550.5	{92 411., 92 704.}	22.8079
11	R0	{2.27349, 14.6338, 1.}	{1.88026, 9.83949, 1.93}	{{1.86868, 9.75343, 2.}, {1.86868, 9.75343, 2.}}	{17.2962, 32.7619, 93.}	inf	19 721.	12 415.5	{12 063.2, 12 474.4}	37.0444	0.201744	newly	8893.	8198.39	{7998., 8355.}	7.95783	0.0361461	Reff	0.854264	0.83405	{0.829807, 0.836883}	2.4385	ar	85 805.	76 043.7	{75 385., 76 182.}	11.3761
12	R0	{2.77261, 14.1894, 0.1}	{2.73108, 15.6124, 0.}	{{2.73108, 15.6124, 0.}, {2.73108, 15.6124, 0.}}	{1.4977, 10.0288, 100.}	inf	27 138.	26 305.9	{26 172.5, 26 444.5}	3.06612	0.0135289	newly	12 745.	11 252.4	{11 084., 11 382.}	11.7113	0.0541158	Reff	0.865526	0.877235	{0.873555, 0.880541}	1.3528	ar	92 508.	91 859.8	{91 727., 91 990.}	0.700707
13	R0	{2.65402, 19.3935, 2.}	{1.78127, 9.10883, 1.01}	{{1.77826, 9.06807, 1.}, {1.77826, 9.06807, 1.}}	{32.8839, 53.0315, 49.5}	inf	25 765.	11 148.7	{10 938.6, 11 281.1}	56.7293	0.364241	newly	8948.	8112.11	{7938., 8263.}	9.34164	0.0426446	Reff	0.987375	0.90315	{0.899253, 0.905398}	8.54388	ar	91 184.	72 480.4	{71 979., 72 771.}	20.5119
14	R0	{1.75297, 4.45354, 0.1}	{2.53075, 11.6597, 0.}	{{2.53075, 11.6597, 0.}, {2.53075, 11.6597, 0.}}	{44.3692, 161.808, 100.}	inf	10 727.	23 801.1	{23 579.1, 23 987.6}	121.88	0.346108	newly	15 310.	13 820.1	{13 670., 13 960.}	9.73181	0.0444817	Reff	0.926059	1.00072	{0.995586, 1.00571}	8.06178	ar	71 263.	89 646.8	{89 474., 89 808.}	25.7971
15	R0	{1.99928, 7.1501, 0.1}	{2.64721, 11.9965, 0.}	{{2.64721, 11.9965, 0.}, {2.64721, 11.9965, 0.}}	{32.4083, 67.7812, 100.}	inf	13 996.	24 850.	{24 632.3, 25 044.4}	77.5504	0.249313	newly	12 056.	13 435.1	{13 290., 13 586.}	11.439	0.0470217	Reff	0.80563	0.832216	{0.827474, 0.836997}	3.29998	ar	79 602.	91 010.3	{90 800., 91 202.}	14.3316
16	R0	{2.67398, 10.3134, 1.}	{2.13505, 8.31782, 0.}	{{2.13505, 8.31782, 0.}, {2.13505, 8.31782, 0.}}	{20.1547, 19.3494, 100.}	inf	23 578.	15 974.6	{15 807.4, 16 172.5}	32.2479	0.169093	newly	14 011.	11 851.8	{11 718., 12 001.}	15.4105	0.0727032	Reff	0.705449	0.757496	{0.754044, 0.761128}	7.37785	ar	91 289.	82 980.8	{82 658., 83 278.}	9.10099
17	R0	{2.44634, 16.3226, 0.1}	{1.98131, 11.1772, 1.}	{{1.98131, 11.1772, 1.}, {1.98131, 11.1772, 1.}}	{19.0092, 31.5231, 900.}	inf	22 666.	14 765.	{14 614.3, 14 928.4}	34.8582	0.186154	newly	9419.	8830.31	{8691., 8964.}	6.25003	0.0280571	Reff	0.907103	0.906179	{0.902794, 0.909299}	0.235809	ar	88 710.	79 145.7	{78 874., 79 407.}	10.7816
18	R0	{2.02168, 10.1676, 0.1}	{2.02235, 10.6804, 0.}	{{2.02235, 10.6804, 0.}, {2.02235, 10.6804, 0.}}	{0.0333691, 5.04351, 100.}	inf	15 535.	15 741.3	{15 601.3, 15 870.8}	1.35895	0.00585192	newly	10 392.	10 107.2	{9973., 10 255.}	2.74105	0.0120938	Reff	0.996001	1.00515	{1.00253, 1.0076}	0.918429	ar	80 299.	80 284.5	{79 945., 80 617.}	0.256491
19	R0	{2.89969, 15.009, 0.1}	{2.15677, 10.1363, 0.}	{{2.15677, 10.1363, 0.}, {2.15677, 10.1363, 0.}}	{25.6206, 32.4653, 100.}	inf	28 996.	17 582.4	{17 410.7, 17 765.4}	39.3628	0.217273	newly	12 862.	11 355.1	{11 211., 11 534.}	11.7157	0.0541399	Reff	0.881738	0.874119	{0.870671, 0.877588}	0.864028	ar	93 349.	83 429.1	{83 156., 83 665.}	10.6267
20	R0	{2.68933, 7.34928, 0.1}	{2.91001, 10.5991, 0.}	{{2.91001, 10.5991, 0.}, {2.91001, 10.5991, 0.}}	{8.20564, 44.2194, 100.}	inf	24 920.	28 476.7	{28 254.8, 28 672.3}	14.2725	0.0579342	newly	20 039.	17 138.7	{16 972., 17 311.}	14.4734	0.0679125	Reff	0.765733	0.843366	{0.837836, 0.849207}	10.1385	ar	91 574.	93 393.7	{93 243., 93 534.}	1.98708
21	R0	{2.15352, 9.28074, 0.1}	{2.46355, 11.7396, 0.9}	{{2.48312, 11.958, 0.}, {2.48312, 11.958, 1.}}	{14.3964, 26.4942, 820.}	inf	17 876.	22 613.4	{22 594.3, 23 107.}	26.5016	0.101361	newly	12 361.	12 783.6	{12 573., 12 999.}	3.41914	0.0145433	Reff	0.8829	0.909747	{0.905149, 0.914291}	3.04071	ar	83 845.	88 707.5	{88 646., 89 250.}	5.79944
22	R0	{2.21953, 9.04387, 0.1}	{2.68687, 12.2271, 1.}	{{2.68687, 12.2271, 1.}, {2.68687, 12.2271, 1.}}	{21.0558, 35.1979, 900.}	inf	18 399.	25 320.6	{25 154.3, 25 494.2}	37.6197	0.138674	newly	13 126.	13 424.4	{13 292., 13 539.}	2.28775	0.00981107	Reff	0.851523	0.820441	{0.815324, 0.824887}	3.65012	ar	84 856.	91 413.5	{91 216., 91 564.}	7.7278
23	R0	{2.21987, 14.5347, 1.}	{2.76795, 19.6041, 1.}	{{2.76795, 19.6041, 1.}, {2.76795, 19.6041, 1.}}	{24.6897, 34.8782, 0.}	inf	18 816.	26 367.8	{26 225.4, 26 518.5}	40.1351	0.146542	newly	8717.	8986.96	{8870., 9112.}	3.09694	0.0132253	Reff	0.868968	0.812408	{0.809047, 0.816021}	6.50884	ar	85 067.	92 206.2	{92 083., 92 321.}	8.39247
24	R0	{2.84268, 17.9659, 1.}	{2.56757, 17.005, 0.}	{{2.56757, 17.005, 0.}, {2.56757, 17.005, 0.}}	{9.67771, 5.34863, 100.}	inf	27 580.	24 097.3	{23 883.2, 24 268.8}	12.6275	0.058633	newly	10 377.	9551.06	{9405., 9685.}	7.95933	0.036045	Reff	0.920602	0.89437	{0.890552, 0.898}	2.84947	ar	92 836.	90 112.1	{89 921., 90 271.}	2.9341
25	R0	{2.52706, 10.3679, 0.1}	{2.7499, 15.3124, 0.}	{{2.7499, 15.3124, 0.}, {2.7499, 15.3124, 0.}}	{8.81804, 47.6906, 100.}	inf	22 248.	25 523.2	{25 344.2, 25 703.6}	14.7212	0.0596375	newly	13 481.	10 842.7	{10 718., 10 974.}	19.5706	0.0946036	Reff	0.761075	0.753964	{0.750072, 0.757998}	0.934852	ar	89 623.	92 044.2	{91 881., 92 163.}	2.70154
26	R0	{2.62835, 19.1088, 1.}	{1.99151, 11.327, 1.07}	{{1.98131, 11.1772, 1.}, {1.98131, 11.1772, 1.}}	{24.2295, 40.7236, 7.}	inf	24 689.	14 942.4	{14 607.9, 15 010.4}	39.4775	0.219493	newly	8698.	8825.43	{8691., 8983.}	1.9424	0.00837745	Reff	0.928833	0.905237	{0.901269, 0.908312}	2.54038	ar	90 908.	79 347.6	{78 880., 79 583.}	12.7166
27	R0	{2.76831, 19.4155, 2.}	{2.35025, 15.0823, 3.}	{{2.32014, 14.7828, 3.}, {2.51417, 16.7164, 3.}}	{15.1018, 22.3181, 50.}	inf	25 708.	19 689.1	{19 098.5, 22 045.2}	23.4125	0.116397	newly	8776.	8549.	{8414., 8692.}	2.60278	0.0114848	Reff	0.75287	0.7649	{0.752498, 0.769996}	1.6788	ar	92 175.	87 049.8	{86 413., 89 330.}	5.56031
28	R0	{2.96609, 16.8903, 1.}	{2.0548, 9.05427, 2.}	{{2.0548, 9.05427, 2.}, {2.0548, 9.05427, 2.}}	{30.7235, 46.3936, 100.}	inf	28 896.	14 951.9	{14 769.6, 15 149.6}	48.2562	0.286163	newly	11 093.	10 424.6	{10 285., 10 585.}	6.0256	0.0270182	Reff	0.743925	0.78634	{0.783106, 0.78977}	5.70149	ar	94 024.	81 091.7	{80 817., 81 421.}	13.7543
29	R0	{2.17046, 14.39, 0.1}	{2.28789, 15.2293, 1.03}	{{2.1255, 13.6009, 1.}, {2.315, 15.4658, 1.}}	{6.0316, 7.47747, 930.}	inf	18 683.	20 080.2	{17 554.7, 20 676.4}	9.35167	0.0390353	newly	8778.	8995.93	{8851., 9140.}	2.61255	0.0111734	Reff	0.946993	0.95282	{0.942362, 0.984622}	0.956996	ar	84 001.	85 987.	{82 840., 86 736.}	2.79837
30	R0	{2.88772, 12.4647, 1.}	{2.79531, 12.1249, 2.}	{{2.79531, 12.1249, 2.}, {2.79531, 12.1249, 2.}}	{3.20015, 2.72599, 100.}	inf	28 167.	27 194.2	{26 999.6, 27 347.3}	3.4537	0.015271	newly	14 527.	14 698.	{14 527., 14 834.}	1.27514	0.00549356	Reff	0.836804	0.884921	{0.879742, 0.890234}	5.7501	ar	93 273.	92 452.3	{92 283., 92 632.}	0.879869
31	R0	{2.46831, 8.14308, 0.1}	{2.17292, 6.16453, 0.}	{{2.17292, 6.16453, 0.}, {2.17292, 6.16453, 0.}}	{11.9675, 24.2973, 100.}	inf	22 336.	17 886.3	{17 671.4, 18 077.6}	19.9217	0.0965027	newly	17 282.	17 849.8	{17 651., 18 026.}	3.28573	0.0140234	Reff	0.883087	0.876862	{0.871967, 0.882163}	0.747145	ar	88 762.	83 818.6	{83 570., 84 093.}	5.56932
32	R0	{1.93236, 7.38987, 0.1}	{2.91168, 14.943, 1.94}	{{2.95376, 15.278, 2.}, {2.99911, 15.6207, 2.}}	{51.7902, 104.589, 1840.}	inf	13 853.	28 496.1	{29 075., 29 969.1}	108.749	0.318331	newly	12 032.	12 627.2	{12 516., 12 891.}	6.0994	0.0259254	Reff	0.884615	0.886258	{0.88128, 0.893221}	0.613583	ar	77 654.	92 591.7	{93 577., 94 150.}	20.2035
33	R0	{2.82251, 17.6954, 1.}	{2.51197, 15.0802, 2.97}	{{2.45256, 14.4765, 3.}, {2.51743, 15.191, 3.}}	{11.0455, 14.7788, 197.}	inf	27 972.	23 510.6	{22 590., 23 766.6}	15.9903	0.0758983	newly	10 722.	10 725.1	{10 571., 10 884.}	1.00354	0.00433811	Reff	1.00304	1.01039	{0.977675, 1.02293}	1.87875	ar	92 745.	89 401.5	{88 570., 89 682.}	3.61444
34	R0	{1.87083, 12.3994, 1.}	{2.1347, 15.9133, 3.99}	{{2.07206, 15.0886, 4.}, {2.15611, 16.1983, 4.}}	{14.3811, 28.9225, 299.}	inf	12 842.	17 613.7	{16 600.4, 18 108.7}	37.8814	0.139907	newly	7135.	7693.51	{7588., 7810.}	8.0391	0.0336017	Reff	1.01449	1.02537	{1.01061, 1.03215}	1.27847	ar	76 282.	82 890.5	{81 502., 83 675.}	8.96117
35	R0	{2.95779, 16.3003, 1.}	{2.49296, 12.2747, 0.01}	{{2.32891, 10.9956, 0.}, {2.52368, 12.5037, 0.}}	{15.7154, 24.6965, 99.}	inf	29 124.	23 218.3	{20 826.2, 23 846.8}	20.2779	0.0989274	newly	12 158.	12 834.	{12 638., 12 983.}	5.56004	0.0234716	Reff	1.01659	0.995255	{0.972217, 1.00404}	2.09891	ar	93 873.	89 121.6	{86 803., 89 748.}	5.06156
36	R0	{2.18718, 14.261, 3.}	{1.93528, 10.7589, 3.02}	{{1.85376, 9.90957, 3.}, {1.92538, 10.6367, 3.}}	{12.5451, 26.0664, 2.66667}	inf	18 672.	13 987.7	{12 723.3, 14 024.}	26.8424	0.13635	newly	8858.	8683.05	{8518., 8861.}	2.21551	0.00981671	Reff	0.915641	0.914176	{0.911347, 0.918872}	0.431679	ar	84 379.	77 548.5	{75 281., 77 932.}	8.528
37	R0	{2.44912, 9.31118, 0.1}	{1.98462, 6.26542, 0.}	{{1.98388, 6.25659, 0.}, {1.9862, 6.28417, 0.}}	{18.966, 32.7108, 100.}	inf	21 427.	13 903.3	{13 708.6, 14 076.8}	35.113	0.187866	newly	14 344.	13 416.5	{13 241., 13 600.}	6.46619	0.029058	Reff	0.796601	0.802602	{0.797288, 0.807646}	0.775519	ar	88 442.	79 231.9	{78 907., 79 499.}	10.4137
38	R0	{2.05949, 5.72514, 0.1}	{2.74056, 10.6768, 0.}	{{2.74056, 10.6768, 0.}, {2.74056, 10.6768, 0.}}	{33.0698, 86.489, 100.}	inf	16 011.	26 155.7	{25 937.4, 26 374.7}	63.3609	0.21314	newly	16 782.	15 653.4	{15 488., 15 796.}	6.72524	0.0302487	Reff	0.827297	0.831586	{0.82648, 0.836258}	0.572313	ar	81 587.	91 961.8	{91 796., 92 149.}	12.7163
39	R0	{1.75505, 8.52404, 0.1}	{1.87927, 9.84456, 1.}	{{1.87927, 9.84456, 1.}, {1.87927, 9.84456, 1.}}	{7.07787, 15.4917, 900.}	inf	10 952.	13 205.4	{13 032.3, 13 365.4}	20.5752	0.0812383	newly	8590.	9148.2	{9009., 9306.}	6.49825	0.0273121	Reff	0.957748	0.982752	{0.979136, 0.986034}	2.6107	ar	70 925.	76 083.4	{75 676., 76 485.}	7.27299
40	R0	{2.24724, 13.7861, 1.}	{2.04871, 11.6322, 3.}	{{2.04871, 11.6322, 3.}, {2.04871, 11.6322, 3.}}	{8.8346, 15.6236, 200.}	inf	18 516.	15 518.2	{15 322.9, 15 683.5}	16.1905	0.076721	newly	8935.	8779.81	{8642., 8926.}	1.80694	0.00794073	Reff	0.839861	0.842808	{0.840084, 0.845498}	0.365426	ar	85 257.	80 970.2	{80 674., 81 242.}	5.02809
41	R0	{1.77635, 8.62406, 0.1}	{1.96373, 10.9366, 4.}	{{1.96373, 10.9366, 4.}, {1.96373, 10.9366, 4.}}	{10.5483, 26.8153, 3900.}	inf	11 281.	14 374.4	{14 217.7, 14 529.5}	27.4209	0.105224	newly	8585.	8729.77	{8593., 8859.}	1.7795	0.00763743	Reff	0.87725	0.88879	{0.885614, 0.891597}	1.31541	ar	72 941.	78 694.3	{78 382., 78 967.}	7.88761
42	R0	{1.80397, 5.4932, 1.}	{2.36298, 10.1198, 2.}	{{2.36298, 10.1198, 2.}, {2.36298, 10.1198, 2.}}	{30.988, 84.2248, 100.}	inf	10 692.	20 708.6	{20 545.6, 20 893.3}	93.6831	0.287082	newly	11 817.	13 195.4	{13 047., 13 320.}	11.6646	0.0479014	Reff	0.8332	0.845603	{0.841044, 0.849257}	1.48867	ar	73 232.	87 270.6	{86 991., 87 519.}	19.1701
43	R0	{2.83096, 16.7077, 0.1}	{2.29704, 13.8037, 0.}	{{2.29055, 13.7413, 0.}, {2.29055, 13.7413, 0.}}	{18.9368, 17.7731, 100.}	inf	27 789.	20 214.1	{19 945.1, 20 260.8}	27.3733	0.139061	newly	11 209.	9966.04	{9835., 10 097.}	11.0889	0.0510678	Reff	0.932207	0.962419	{0.958324, 0.965942}	3.24091	ar	92 740.	86 160.4	{85 842., 86 350.}	7.11492
44	R0	{2.73678, 11.6021, 1.}	{2.99939, 13.7076, 1.}	{{2.99939, 13.7076, 1.}, {2.99939, 13.7076, 1.}}	{9.5957, 18.1478, 0.}	inf	23 763.	27 331.4	{27 154.6, 27 500.5}	15.0168	0.0607557	newly	12 451.	12 534.7	{12 386., 12 675.}	0.908843	0.00392391	Reff	0.638655	0.649084	{0.645117, 0.653315}	1.63291	ar	92 003.	94 039.8	{93 924., 94 161.}	2.21383
45	R0	{2.14235, 6.98601, 0.1}	{2.13505, 8.31782, 0.}	{{2.13505, 8.31782, 0.}, {2.13505, 8.31782, 0.}}	{0.340824, 19.0639, 100.}	inf	15 339.	15 981.7	{15 815., 16 194.3}	4.1898	0.0178077	newly	13 010.	11 865.3	{11 700., 12 020.}	8.79877	0.0400249	Reff	0.718266	0.757384	{0.753096, 0.761223}	5.44616	ar	82 974.	83 003.9	{82 736., 83 285.}	0.20768
46	R0	{2.23409, 9.21289, 0.1}	{2.53075, 11.6597, 0.}	{{2.53075, 11.6597, 0.}, {2.53075, 11.6597, 0.}}	{13.2788, 26.5587, 100.}	inf	19 256.	23 781.5	{23 576.3, 24 003.2}	23.5016	0.0916608	newly	14 108.	13 815.6	{13 634., 14 020.}	2.07698	0.00913674	Reff	0.983357	1.00095	{0.995113, 1.00591}	1.78949	ar	85 163.	89 625.2	{89 415., 89 817.}	5.23958
47	R0	{2.12258, 8.15265, 0.1}	{2.49494, 10.8657, 1.64}	{{2.19024, 8.25876, 1.}, {2.6266, 11.8519, 2.}}	{17.5427, 33.2785, 1540.}	inf	17 054.	22 903.	{17 891.4, 24 838.3}	34.2968	0.124202	newly	13 477.	13 828.9	{13 593., 14 416.}	2.62262	0.0111352	Reff	0.871829	0.893715	{0.847897, 0.918768}	3.92162	ar	82 690.	88 880.6	{84 120., 90 753.}	7.48654
48	R0	{2.71771, 10.4636, 0.1}	{2.26582, 8.12562, 0.}	{{2.26582, 8.12562, 0.}, {2.26582, 8.12562, 0.}}	{16.6275, 22.344, 100.}	inf	26 340.	19 515.1	{19 329.8, 19 675.2}	25.911	0.130259	newly	16 548.	15 424.	{15 240., 15 582.}	6.79254	0.0305675	Reff	0.927228	0.901536	{0.896667, 0.90659}	2.77085	ar	91 716.	85 638.7	{85 404., 85 849.}	6.62626
49	R0	{2.44369, 6.66477, 0.1}	{2.77394, 8.24535, 0.}	{{2.77394, 8.24535, 0.}, {2.77394, 8.24535, 0.}}	{13.5144, 23.7155, 100.}	inf	18 124.	22 375.1	{22 190.4, 22 558.}	23.4559	0.0915019	newly	14 957.	15 405.8	{15 235., 15 558.}	3.00047	0.0128239	Reff	0.592717	0.569201	{0.563787, 0.573237}	3.96742	ar	88 827.	92 250.4	{92 113., 92 386.}	3.85399
50	R0	{2.77383, 17.9775, 0.1}	{2.68049, 16.9709, 0.5}	{{2.64334, 17.2462, 0.}, {2.88523, 18.2127, 1.}}	{6.78013, 6.82148, 500.}	inf	25 584.	24 199.7	{24 049., 26 772.5}	9.05469	0.0474072	newly	9271.	9297.1	{8983., 9627.}	2.64804	0.0116695	Reff	0.747852	0.742925	{0.698269, 0.777147}	4.68702	ar	92 334.	90 719.	{90 699., 93 280.}	2.55635
